# An open-source and spatially diverse synthetic population dataset for agent-based modelling and microsimulation in Ireland

**DOI:** 10.1016/j.dib.2025.111611

**Published:** 2025-05-01

**Authors:** Seán Caulfield Curley, Karl Mason, Patrick Mannion

**Affiliations:** School of Computer Science, University of Galway, Galway, Ireland

**Keywords:** Microsimulation, Agent-based modelling, Synthetic population, Ireland

## Abstract

Spatial microsimulations, where simulation units represent people or households in a small area, are extremely useful for modelling a wide range of socio-economic scenarios at a fine scale. The characteristics of individuals in these simulations' populations need to accurately represent the real characteristics of the target area to model realistic scenarios. However, individual-level data is not available for the vast majority of populations, Ireland included, due to privacy concerns. Thus, a representative synthetic population for the Republic of Ireland is needed. The data from four methods of generating synthetic populations at the Electoral Division level are given in this paper. Realistic individuals are created by sampling from the Central Statistics Office (CSO) Labour Force Survey. Spatial heterogeneity is achieved by matching the aggregate counts of individuals' characteristics to those from the CSO Census Small Area Population Statistics. Individuals are assigned six characteristics: age group, sex, marital status, house size, primary economic status, and highest level of education achieved.

## Graphical abstract

**Figure:**
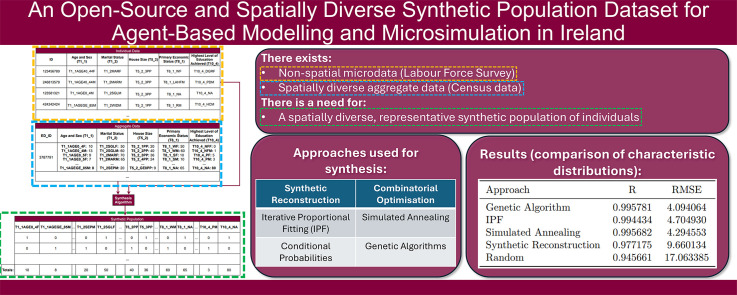

Specifications Table

**Table:** 

Subject	Computer Sciences
Specific subject area	Synthetic Population of Irish people with socioeconomic characteristics
Type of data	Tables of people in Electoral Divisions and their characteristics (.csv & .pkl) Code to generate populations (.py & .R)
Data collection	Iterative Proportional Fitting (IPF) [1], Synthetic Reconstruction, Genetic Algorithms, and Simulated Annealing were implemented in Python and/or R. They were used to match aggregate characteristic counts from the census with realistic individuals from the Labour Force Survey.
Data source location	Central Statistics Office (data available from data.cso.ie) Labour Force Survey (data available from https://www.ucd.ie/issda/data/lfs/)
Data accessibility	Repository name: Zenodo Data identification number: 10.5281/zenodo.14802378 Direct URL to data: 10.5281/zenodo.14802378 Instructions for accessing these data: Clone the GitHub repository and open results with any CSV viewer
Related research article	None

## 1 Value of the Data

• This synthetic population data will enable agent-based modelling and microsimulations for a wide range of socioeconomic scenarios in Ireland including housing, education and employment.
• The spatial heterogeneity of the data will allow a researcher to investigate issues at a local level, not just at the provincial or national scale. For example, a local council could use the population to analyse their local areas' age and marital status distributions when looking for the best place to build a new civil registration service.
• The populations created by IPF and Synthetic Reconstruction do not contain real people which preserves privacy. This means researchers do not have to go through the process of getting approved to access the data of real people to be able to perform experiments with realistic populations.
• The data generated by the Genetic Algorithm and Simulated Annealing approaches is matched to real respondents from the Labour Force Survey. This allows for the usage of any of the other unconstrained characteristics that a person in the synthetic population may have such as hours worked, number of jobs held or methods for seeking employment.
• The attached population generation code is generalised to the choice of characteristics meaning a researcher with access to different microdata could easily generate a population for their purposes. For example, a researcher could use the National Transport Survey instead of the Labour Force Survey as the microdata if they wanted to simulate public transport usage for all of the Electoral Divisions in Ireland.

## 2 Background

In microsimulation, simulation units represent small-scale real-world entities like people and households. In spatial microsimulation, units are initialised in a geographical location and are assigned attributes based on those observed in that geographical area. This is useful for analysing local issues including health [2], agriculture [3], and public transport [4]. In order to correctly assign realistic attributes to an area's people/households, methods for synthetic population generation like Iterative Proportional Fitting (IPF), Simulated Annealing, etc., can be applied. National-level populations have been created for countries like the United States [5], Sweden [6], and Canada [7] but no open-source populations have been released for Ireland yet.

To generate a spatially diverse synthetic population, one needs aggregate data for each target area, such as that which is collected in the Census. A synthetic population could be generated from this aggregate data alone [8] but the data must be highly multivariate to form realistic individuals. In order to create synthetic populations with realistic joint distributions of characteristics without linked aggregate data, spatial microsimulation methods utilise a small, representative sample of the desired population to aid the generation process.

## 3 Data Description

### 3.1 Populations

For both the Synthetic Reconstruction and IPF approaches, a CSV file and a serialised pickle file for each Electoral Division is given in the results folder. CSVs are human-readable and intuitive but also storage-inefficient. Pickle files are included to allow python users to load a population as an object easily. Each file is named according to its Electoral Division's ID from the Small Area Population Statistics (SAPS) data. Population files are not included for the approaches which use individuals from the microdata directly (Simulated Annealing and Genetic Algorithms) because of privacy concerns. However, a researcher with access to the Labour Force Survey microdata can quickly generate the populations using the attached code.

Each row in a population file represents an individual. The columns of the CSV correspond to a person's age group and sex, marital status, household size, primary economic status and highest level of education achieved. The complete list of characteristic categories and their corresponding characteristics are given in Table 1. Each characteristic is assigned the same name as it has in the Small Area Population Statistics. For example, the primary economic status of “Student” is in Table T8_1 of the SAPS and has the value “S” so it is given the name “T8_1_S”. The ID of individuals sampled from the Labour Force Survey are also attached when using the Simulated Annealing or Genetic Algorithm approaches. This allows for the inclusion of any of the individual's other characteristics from the Labour Force Survey.

**Table 1:** Constraint characteristics after pre-processing to achieve a match between LFS and SAPS data.

Characteristic (SAPS Table Prefix)	Categories	Code
Age Group and Sex (T1_1)	5 Year Age Groups e.g. (0-4, 5-9, ..., ≥85) followed by Male (M) or Female (F)	T1_1AGE{*lower_age*}_ {*upper _age*}{*Sex*}
Marital Status (T1_2)	Single	T1_2SGL{*Sex*}
	Married	T1_2MAR{*Sex*}
	Widowed	T1_2WID{*Sex*}
	Separated	T1_2SEP{*Sex*}
Housing Size (T5_2)	1 person	T5_2_1PP
	2 people	T5_2_2PP
	3 people	T5_2_3PP
	4 people	T5_2_4PP
	5 people	T5_2_5PP
	6 people	T5_2_6PP
	7 people	T5_2_7PP
	≥8 people	T5_2_GE8PP
Primary Economic Status (T8_1)	At work	T8_1_W{*Sex*}
	Student	T8_1_S{*Sex*}
	Looking After Home/Family	T8_1_LAHF{*Sex*}
	Retired	T8_1_R{*Sex*}
	Unable to Work Due to Permanent Sickness or Disability	T8_1_UTWSD{*Sex*}
	Other	T8_1_OTH{*Sex*}
	Unemployed	T8_1_UNE{*Sex*}
	Not Applicable (for children, etc.)	T8_1_NA
Highest Level of Education Achieved (T10_4)	No Formal Education	T10_4_NF{*Sex*}
	Primary Education	T10_4_P{*Sex*}
	Lower Secondary	T10_4_LS{*Sex*}
	Upper Secondary	T10_4_US{*Sex*}
	Post-Leaving Certificate	T10_4_PLC{*Sex*}
	Higher Certificate	T10_4_HC{*Sex*}
	Undergraduate Degree	T10_4_DGR{*Sex*}
	Postgraduate Degree	T10_4_PD{*Sex*}
	Doctorate	T10_4_D{*Sex*}
	Not Stated	T10_4_NS{*Sex*}
	Not Applicable (for children, students, etc.)	T10_4_NA

### 3.2 Code

The outline of each of the main attached scripts is as follows:

• Pre-Processing: csv_constraint_loader.py reads the raw SAPS CSV data, pre-processes it and transforms it into the desired format for population generation.
• Synthetic Reconstruction: conditional_probabilities.py creates a synthetic population for each ED in Ireland
• IPF:
  ○ ipf_initial_matrix.py creates a cross-tabulation of characteristics from the LFS microdata which is needed as the initial seed matrix for IPF.
  ○ ipf.R creates a synthetic population for each ED in Ireland using the mipfp package in R [9]
  ○ ipf_results_to_pkl.py puts the IPF results in the same format as the other approaches.
  ○ generate_population_households.py can be run with either of the arguments “genetic_algorithms” or “simulated_annealing”. It will create a synthetic population for each ED in Ireland using the chosen approach and can be used if households, rather than people, are desired as the simulation units.

## 4 Experimental Design, Materials and Methods

### 4.1 Input data

Our aim is to create a representative synthetic population for each of the 3440 Electoral Divisions (EDs) in Ireland. Six characteristics were chosen to represent an individual. Those characteristics are age, sex, marital status, house size, principal economic status and highest level of education achieved. This follows the “more the merrier” method [10] of choosing constraint variables as these are all of the characteristics which have some sort of match between the two sources of data. Constraining the populations with as many variables as are available allows for the most spatial diversity possible.

#### 4.1.1 Aggregate statistics

EDs are the smallest legally defined administrative area in the Republic of Ireland. The median population of the EDs in the 2022 Census is 675 while the largest ED, Blanchardstown-Blakestown, has a population of 43,905. The smallest ED, Castletown (County Clare), has a population of 71. For statistical disclosure reasons, the CSO publishes data for 3420 of the 3440 EDs. However, 3 pairs of EDs, namely Castletown North and South (County Louth), Dundalk Rural North and South, and St. Mary's East and West (County Cork), share the same ID and so their statistics are summed for our purposes.

The Small Area Population Statistics (SAPS)^1^ are derived from the Census which is performed by the CSO approximately every 5 years. On Census night, all people in Ireland are counted. However, in some of the CSO's published results 3 or more subsets of the population on the night are considered. For example, table T5_2 “People in Private Households by Size” only includes individuals who are usually resident in Ireland. Furthermore, T5_2 also includes individuals who were not present on Census night but who do normally reside in the ED in which they were counted. Non-private housing, such as hotels, prisons or nursing homes, also presents a challenge as people in communal housing are counted in tables such as T1_1 “Population by Age and Sex” but not in those for housing size.

Some questions in the Census are optional or are only meant to be answered by a certain subset of the present population. For example, individuals who identify their primary economic status as a student are not included in the table T10_4 “Highest Level of Education Completed” (neither are children or those in other forms of training). For approaches like IPF, all characteristics are required to sum to the same total so the data had to be re-scaled before synthesis was performed. Therefore, for tables where answering was optional an additional category of “*table_name*_NA” was added. All tables were then re-scaled using an approach based on Bresenham's Line Drawing Algorithm [11] to total the same as the number of people usually resident in the ED (the total of the T5_2 table). Re-scaling in this manner assumes similarity between the distribution of characteristics between the following pairs of populations:

• Those usually resident who were present on Census night and those usually resident who were not present on Census night.
• Tourists (present on Census night) and non-tourists.
• Those in communal living quarters and those in private households.

The final assumption in particular may not be realistic. This is because e.g., nursing homes are predominantly inhabited by older retirees and prisons are largely inhabited by males [12]. However, there is an absence of more fine-grained data for the characteristics of individuals in non-private homes. Therefore, users of the final population dataset are encouraged to consider how this re-scaling may affect the socio-economic outcomes of their simulations, and to adjust as necessary.

#### 4.1.2 Individual data

Approaches like Simulated Annealing require a sample of individuals or households to form the synthetic population from. Anonymised Census microdata files are available but only from 2016 at the most recent. Similarly, the previously used Living in Ireland survey is only available up to 2001. A more recent dataset is available in the form of the Labour Force Survey (LFS) where the most recent records are from the third quarter of 2023. 31,160 individuals are included in the 2023 Q3 dataset. The dataset contains a range of information on each individual's demographic, employment status, education and dwelling data along with detailed insights into the specifics of their work. The level of overlap between the LFS and SAPS data means that a tightly constrained population can be achieved.

There is not, however, a one-to-one correspondence between the categories of the characteristics shared by both the LFS and the SAPS data. Therefore, a number of preprocessing steps were taken to match equivalent tables together. In the SAPS data, the number of individuals at each year of age from 0 to 19 are given separately. These were combined into 5-year age groups in keeping with the LFS and the data for adults in the SAPS. Separated and divorced individuals are counted separately in the SAPS but are counted in the same category in the LFS. Types of unemployment (looking for first regular job, short- and long-term unemployed) are also counted individually in the SAPS but given under the broad header of “Unemployed” in the LFS. In both of these cases, the more detailed SAPS categories were summed and given a new codename corresponding to their title in the LFS. Finally, the highest levels of education achieved are counted approximately at the National Framework of Qualifications level in the SAPS data while the LFS data uses the International Standard Classification of Education. Coherence between education levels was achieved by converting the characteristic of those with an ISCED level 4 qualification to a new label of “Post Leaving Certificate” and those with an ISCED level 6 qualification to a new label of “Undergraduate Degree”. The full list of characteristics and their corresponding categories are given in Table 1.

### 4.2 Methods

**Fig. 1:**
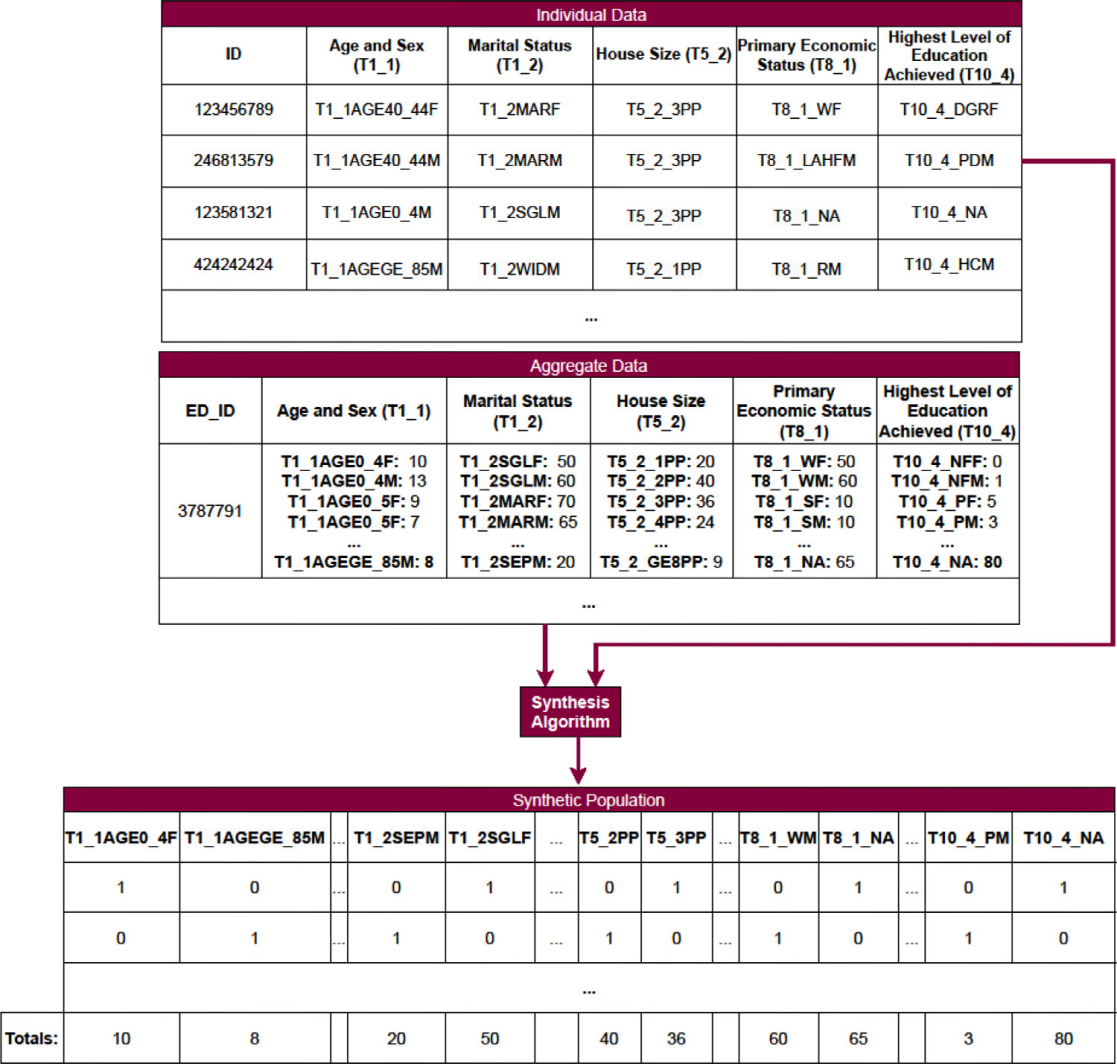
Flowchart illustrating the format of both the input and output data. The individual and ED values are created for the example and do not represent any real people or places.

We use a number of different methods for generating our synthetic populations all of which use the LFS to guide their synthesis. However, some methods use respondents from the LFS directly while others only use the distributions of characteristics seen in the LFS. These methods are referred to as Combinatorial Optimisation (CO) and Synthetic Reconstruction (SR), respectively. In general, CO methods allow us to synthesise households more easily than SR (by taking entire households from the LFS). However, SR methods provide privacy benefits as none of the individuals in the final synthetic population are real. This means that the population dataset can be published directly rather than only the code required to create the dataset being publishable. All algorithms are implemented in Python except for IPF which is implemented in R to take advantage of the “mipfp” package [9]. The overall structure of both the individual and aggregate data as well as the format of the final synthetic population dataset is given in Fig. 1.

#### 4.2.1 Combinatorial optimisation

##### 4.2.1.1 Simulated annealing

Simulated Annealing (SA) is a general optimisation technique which is used in many fields (including VLSI design [13] and solar cell model parameter estimation [14]) and which is particularly popular in the creation of synthetic populations. For example, Williamson et al. [15] used SA to form spatially diverse populations for two areas in Leeds and found a strong goodness-of-fit between the generated populations and the target areas' population statistics. In SA, we take a random sample of the correct number of households from the LFS and evaluate the sample's fit in relation to the aggregate counts of constraining characteristics from the spatial dataset (the SAPS). Then, a number of households are chosen randomly to potentially be swapped out for an equally sized random sample from the microdata (the LFS). The number of households chosen for swapping is dictated by a parameter called “temperature”. The temperature used is decreased after each iteration. In our case, 85% of the previous iteration's temperature value is used for the current iteration. If the goodness-of-fit of the population with the new households from the microdata is better than the previous goodness-of-fit, the households are swapped. This is repeated until an acceptable fit is achieved. There is an exception which allows households to be swapped even if the goodness-of-fit of the population with the new households is worse. The probability of accepting a worse solution is given by:

(1) $$P\left(acceptance\right)=e^{-\frac{\Delta E}{kT}}$$

where ΔE represents the difference in error from the current iteration to the last and k is a constant which can be set to 1 for our purposes. T denotes the current temperature value. Evidently, as the temperature decreases with more and more iterations, the probability of acceptance also decreases. This means that the algorithm can explore the search space initially and then narrow in on a “good” region to refine the final solution.

##### 4.2.1.2 Genetic algorithms

Like Simulated Annealing, Genetic Algorithms (GAs) are a general optimisation technique utilised in a wide array of research areas (including HVAC control [16] and building design [17]). GAs were also used along with SA in the study by Williamson et al. [15] of the small areas in Leeds and again, a strong match between the areas' statistics and the generated population was found. A GA is a type of evolutionary algorithm which is inspired by biological operators such as selection, crossover and mutation. For population synthesis, a GA operates as follows (Algorithm 1):

**Algorithm 1:** Genetic Algorithm for synthetic population generation.

Randomly sample a number of initial synthetic populations from the microdata and calculate a goodness-of-fit measure for each. **for** a defined number of generations **do**	
	**Generate pairs of parents** for the next generation using tournaments. In a tournament, a random sample of the potential populations are chosen and the one with the best fitness is returned. The size of a tournament in this experiment was 10. **Create two children from the parents using crossover.** Here, crossover is performed by choosing a number (controlled by a crossover rate parameter) of people/households and then swapping them between the two parent populations. **Mutate each of the children.** To mutate the synthetic populations, people/households are removed at random and replaced by people/households from the microdata. The level of mutation is controlled by the mutation rate parameter. The current generation’s children become the next generation’s parents
**end for** Return the population with the best fitness	

The number of initial populations used in this experiment was 100. Tournament size was set to 10 while the crossover and mutation rates were 0.7 and 0.05 respectively. Elitism was not utilised. Both SA and GAs can generate populations of either households or individuals.

### 4.3 Synthetic reconstruction

#### 4.3.1 Synthetic reconstruction using conditional probabilities

This method works by creating individuals one at a time, one characteristic at a time. The first characteristic considered is sampled using Monte Carlo sampling from a distribution which is proportionally equal to the distribution for that characteristic in the target area aggregate data (SAPS). The next characteristic is assigned by sampling from the conditional distribution of the characteristic from the microdata given that the first characteristic has been assigned already. This step is then repeated for all other characteristics, sampling from increasingly restrictive conditional distributions.

An example of the procedure where the first characteristic chosen was sex could proceed as follows; if the distribution of sexes in an area is 55 % male and 45 % female, an individual will be assigned male with 0.55 probability and female with 0.45 probability. After sampling, the individual was assigned a sex of female and the next characteristic chosen was highest level of education achieved. The distribution of education levels of females only is taken from the microdata. Then, suppose the level of education chosen was postgraduate degree and the next characteristic chosen for sampling was age. Only the distribution of the ages of women with postgraduate degrees in the LFS would be sampled next. Eventually we may end up with a conditional distribution which looks something like P(HouseSize|Retired,Married,Aged70−74,PostgraduateDegree,Female).

This procedure is repeated for all individuals until the correct population size is achieved. Along the way, the order in which the characteristics are sampled can be changed as only the first sampled characteristic will be directly taken from the target area's aggregates. For example, if the distance between the proportional distribution of ages in the synthetic population was straying far from the target area's distribution, age could be sampled first to allow its values to begin aligning more with the area's values. Harland et al. [18] found that Synthetic Reconstruction achieved largely comparable results to SA when they created a synthetic population for the Leeds Metropolitan District Area.

##### 4.3.1.3 IPF

IPF is a ubiquitous method for generating synthetic populations which has been used to generate highly representative populations for many countries, including Canada [7] and the US [5]. It can be performed with or without a sample dataset. In IPF, the target matrix is transformed to proportionally fit a “seed” matrix, but with the row and column totals equal to the target area's marginal distributions of each characteristic. The process is done iteratively, by first multiplying each value of the first dimension by the ratio between its target sum and its current sum. This is repeated for the second dimension, then the third and so on until all dimensions have been scaled once. The whole process is then repeated for each of the dimensions until all of the dimensions totals are equal to the target area's totals. The seed matrix in this case is taken from the LFS where each value is a cross-tabulation of the relevant characteristic categories. This ensures the synthesised population will have a similar joint distribution of characteristics as a realistic population.

### 4.4 Evaluation

#### 4.4.1 Overall evaluation

The median overall Pearson correlations and Root Mean Squared Error (RMSE) values for each approach are given in Table 2:

**Table 2:** Median overall validation results for each algorithm.

Approach	R	RMSE
Genetic Algorithm	0.995781	4.094064
IPF	0.994434	4.704930
.Simulated Annealing	0.995682	4.294553
Synthetic Reconstruction	0.977175	9.660134
Random	0.945661	17.063385

Unsurprisingly, the 2 approaches which sample individuals directly from the microdata (Genetic Algorithms and Simulated Annealing) all create highly representative populations. IPF achieves similar results despite only having access to the area's marginals and the contingency table of the microdata. Synthetic Reconstruction lags a little behind but still achieves approximately 97% average correlation to the aggregate data. A surprising point to note is the relatively competitive performance of random selection. Here, the same number of households were sampled randomly from the microdata as were enumerated in the target ED in the aggregate data. This illustrates that many EDs possess similar overall distributions of characteristics to the national averages.

#### 4.4.2 Evaluation at the electoral division level

In order to validate the above approaches at the lowest scale of aggregation, two metrics for goodness-of-fit suggested by Voas and Williamson [19] are employed.

The first measure is the Pearson goodness-of-fit test. The Pearson statistic is calculated with:

(2) $$X^{2}=\sum \frac{{\left(T_{i}-Ei\right)}^{2}}{E_{i}}$$

where $T_{i}$ and $E_{i}$ are the observed and expected (actual) counts in cell i. The equation is undefined for an expected count of 0 so the authors recommend replacing an $E_{i}$ of 0 with 1.

The second measure are $Z^{2}$ scores. The Z-score for a cell is calculated using the binomial distribution, where the normal distribution is used to approximate the exact binomial value. This approximation leads to difficulty for small values, as a discrete distribution is being modelled as a continuous distribution. A continuity correction factor of 1/2N, where N is the total count for that cell, is included in the Z-score calculation to counteract this. The value is added if the expected proportion is larger than the observed, and subtracted if it is smaller. Z-scores also need correction for zero in the expected proportion. In practice, this is done by replacing the zero proportion with 1/N. The justification for this is included in the paper by Voas and Williamson [19]. The final equation for calculating the Z-score for a cell is then:

(3) $$Z=\frac{\left(t_{i}-p_{i}\right)\pm \frac{1}{2N}}{\sqrt{\frac{p_{i}\left(1-p_{i}\right)}{N}}}$$

where $t_{i}$ is the observed proportion for the cell and $p_{i}$ is the expected proportion for the cell. The continuity correction factor is not included if $p_{i}$ is 0. The $Z^{2}$ score for a table can then be calculated by summing the square of the Z-scores.

Both $X^{2}$ and $Z^{2}$ are then tested for their fit using a $\chi^{2}$ test. Under the correct conditions, both distributions will have a sampling distribution approximately equal to that of a $\chi^{2}$ distribution with the same number of degrees of freedom as the total number of cells (characteristic values) in the table. Thus, $\chi^{2}$ tests a null hypothesis that the observed and expected counts/proportions come from the same distribution. The critical value is calculated according to the confidence level (here α=0.05) and if the $X^{2}$ or $Z^{2}$ score exceeds this, we reject the null hypothesis.

**Fig. 2:**
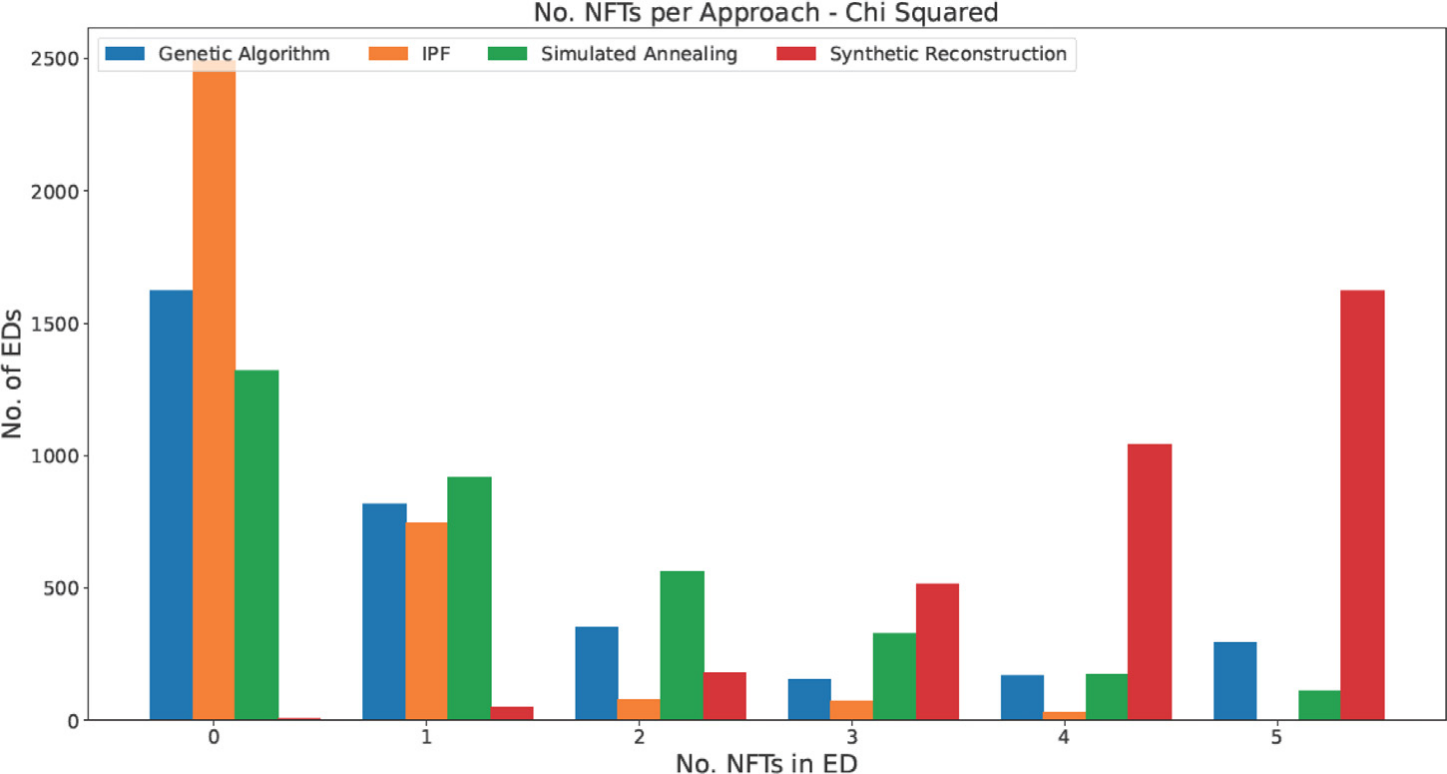
The number of EDs per number of non-fitting tables using Pearson's goodness-of-fit test.

**Fig. 3:**
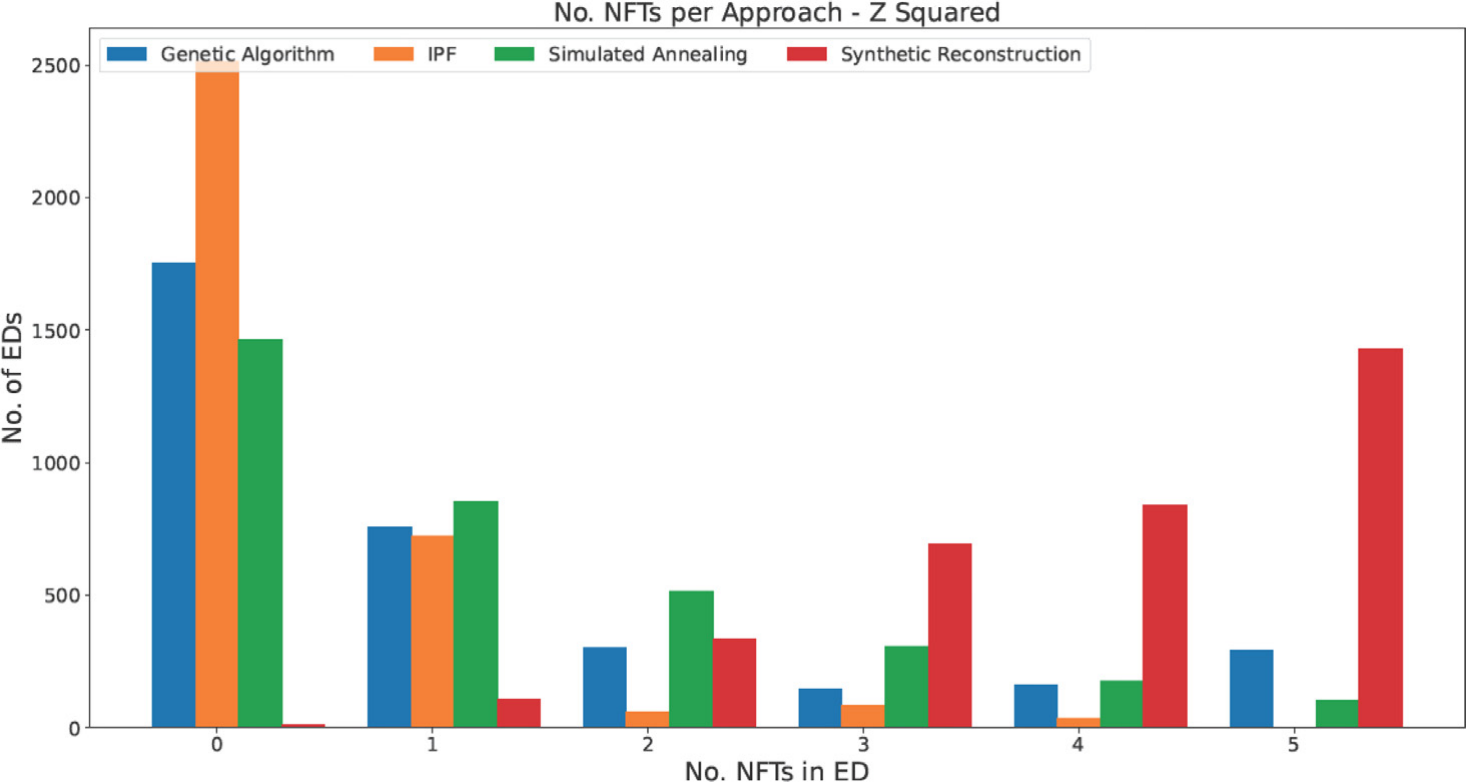
The number of EDs per number of non-fitting tables using a $Z^{2}$ test.

Validation of the above approaches using both $X{}^{2}$ and $Z^{2}$ tests is given in Fig. 2, Fig. 3. The values presented are the number of Non-Fitting Tables (NFTs) per ED. NFTs are a count of how many synthetic characteristic tables in an ED (of a possible 5) failed, i.e., had a $X^{2}$ or $Z^{2}$ exceeding the test's critical value. There is far more heterogeneity in the results of these tests than those from the Pearson correlation or RMSE in Table 2. Approximately 2500 of the 3417 populations generated by IPF fit every characteristic table for their ED and IPF produces no totally non-fitting populations. As opposed to its relatively acceptable performance as calculated in Table 2, Synthetic Reconstruction lags far behind the other approaches in this stage of testing. It produces more completely non-fitting populations than any other single level of fitness. Simulated Annealing and GAs, the two approaches which build populations with individuals from the microdata directly, achieve relatively comparable results to one another. GAs do produce more perfectly fitting populations but also produces over double the number of ED's populations with 5 NFTs.

**Fig. 4:**
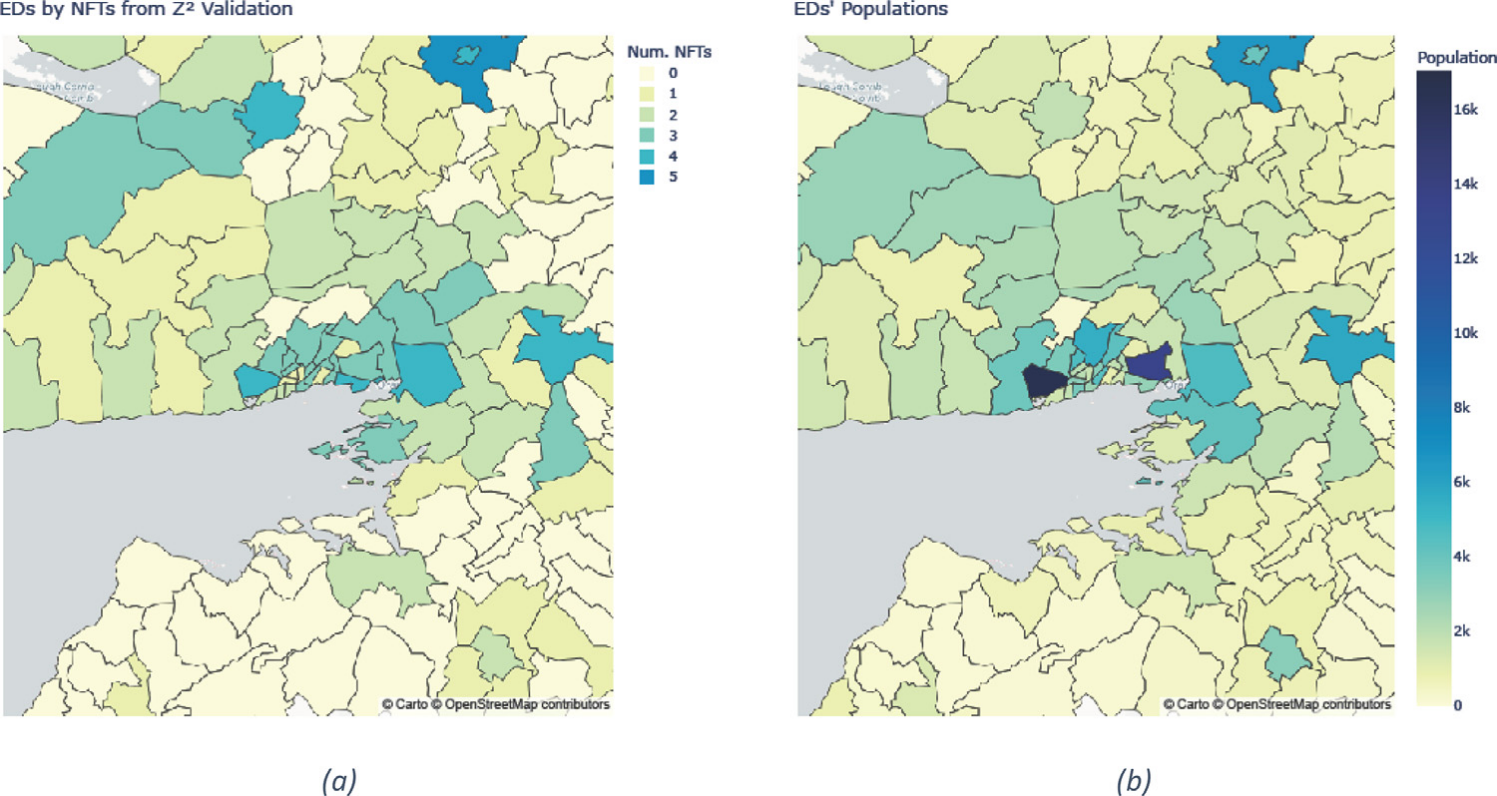
(a) The number of NFTs generated by Simulated Annealing and (b) the total population, for EDs in Galway City and the surrounding area.

The potential reasoning behind an ED's population being synthesised correctly or not is a point of interest. De mooij et al. [20] in their study of the Zuid-West district of The Hague in the Netherlands, suggest that there was an inverse correlation between the population density of a neighbourhood and its error. The rounding of the source data due to privacy concerns is proposed as a possible reason for this link. To investigate if a similar phenomenon was happening here, the number of NFTs along with the total population was plotted for a number of EDs in Galway City and the surrounding areas (Fig. 4). Results for the NFTs plot are taken from the Simulated Annealing approach scored using $Z^{2}$ testing.

Fig. 4 is surprising in that more populous areas, rather than lower density areas, appear prone to errors. The two largest EDs in Galway City: Barna and Ballybane, are modelled with 4 and 3 NFTs respectively. A possible reason for this correlation is that an increase in the required population size gives more opportunities for the rescaling procedure outlined in the Aggregate Statistics Section to fail. Tourists, people living in nursing homes and students in communal accommodation are all more likely to be present in large urban EDs than small rural EDs. Another possible reason for the correlation, especially for the Combinatorial Optimisation approaches, is that a large ED has a higher probability of having someone with an unusual combination of characteristics enumerated there.

#### 4.4.3 Evaluation by characteristic

In order to gain a closer insight into the goodness-of-fit achieved by each approach, the R and RMSE values for each of the individual characteristics was calculated also. The R values are given in Table 3 while the RMSE values are given in Table 4.

**Table 3:** The median R values for each algorithm for each of the tested approaches.

Approach	Age and Sex	Marital Status	House Size	Primary Economic Status	Highest Level of Education Achieved
Genetic Algorithm	0.925286	0.999125	0.998439	0.99713	0.992839
IPF	0.932744	0.996996	0.996126	0.990208	0.996562
Simulated Annealing	0.930837	0.999436	0.999584	0.99726	0.992104
Synthetic Reconstruction	0.748467	0.986452	0.944561	0.983002	0.983746
Random	0.435572	0.978551	0.867719	0.968089	0.953317

**Table 4:** The median RMSE values for each algorithm for each of the tested approaches.

Approach	Age and Sex	Marital Status	House Size	Primary Economic Status	Highest Level of Education Achieved
Genetic Algorithm	2.778889	3.122499	3.724916	3.794733	5.752846
IPF	3	5.97913	5.766281	6.65332	3.952094
Simulated Annealing	2.924988	2.692582	2.150581	4.06612	6.21059
Synthetic Reconstruction	5.338539	11.19152	19.62779	9.859006	8.170796
Random	7.505553	22.80077	31.81195	16.6333	17.37815

Evidently, Genetic Algorithms, IPF and Simulated Annealing all perform similarly with a high goodness-of-fit across all characteristics. Again, Synthetic Reconstruction cannot achieve the same scores as the other algorithms and it particularly struggles to capture the variance of the Age and Sex characteristic. This is the characteristic with by far the most possible values which could suggest an inversely proportional relationship between the breadth of a characteristic and the accuracy to which that characteristic can be modelled. The weaknesses of random selection of households are particularly evident in Table 4, where the median RMSE for 3 of the 5 characteristics is approximately double that of the next worst approach. This could imply that the relatively acceptable R score illustrated in Table 2 is more of an artifact of comparison across many values which are in the same order of magnitude rather than a truly representative synthetic population.

**Fig. 5:**
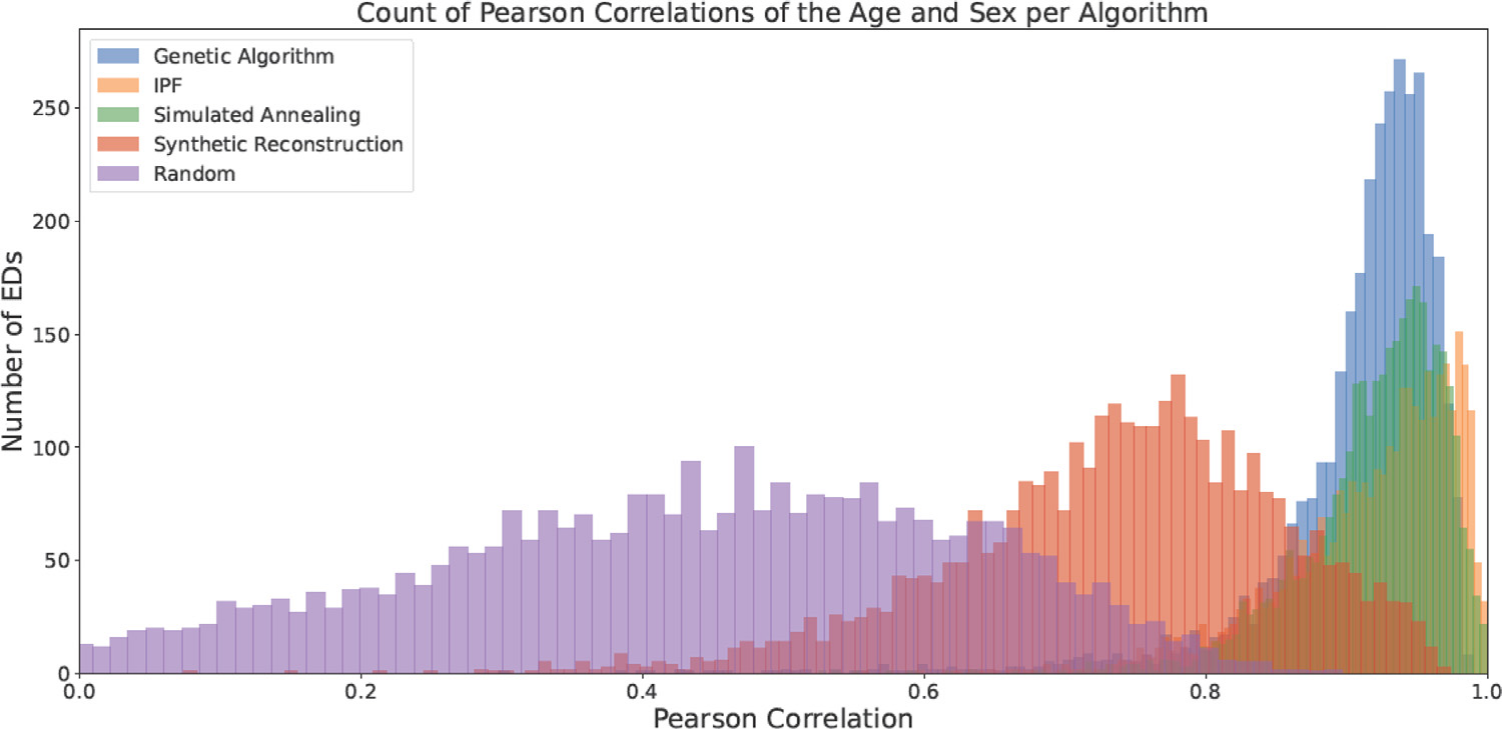
Counts of the Pearson Correlation per algorithm for Age and Sex.

**Fig. 6:**
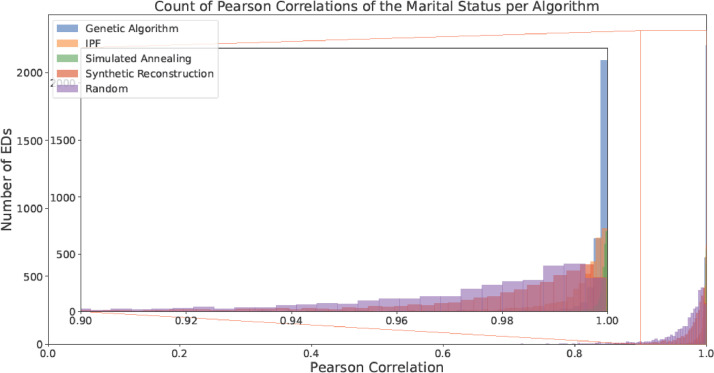
Counts of the Pearson Correlation per algorithm for Marital Status (with a focus on the area with > 0.9 correlation).

**Fig. 7:**
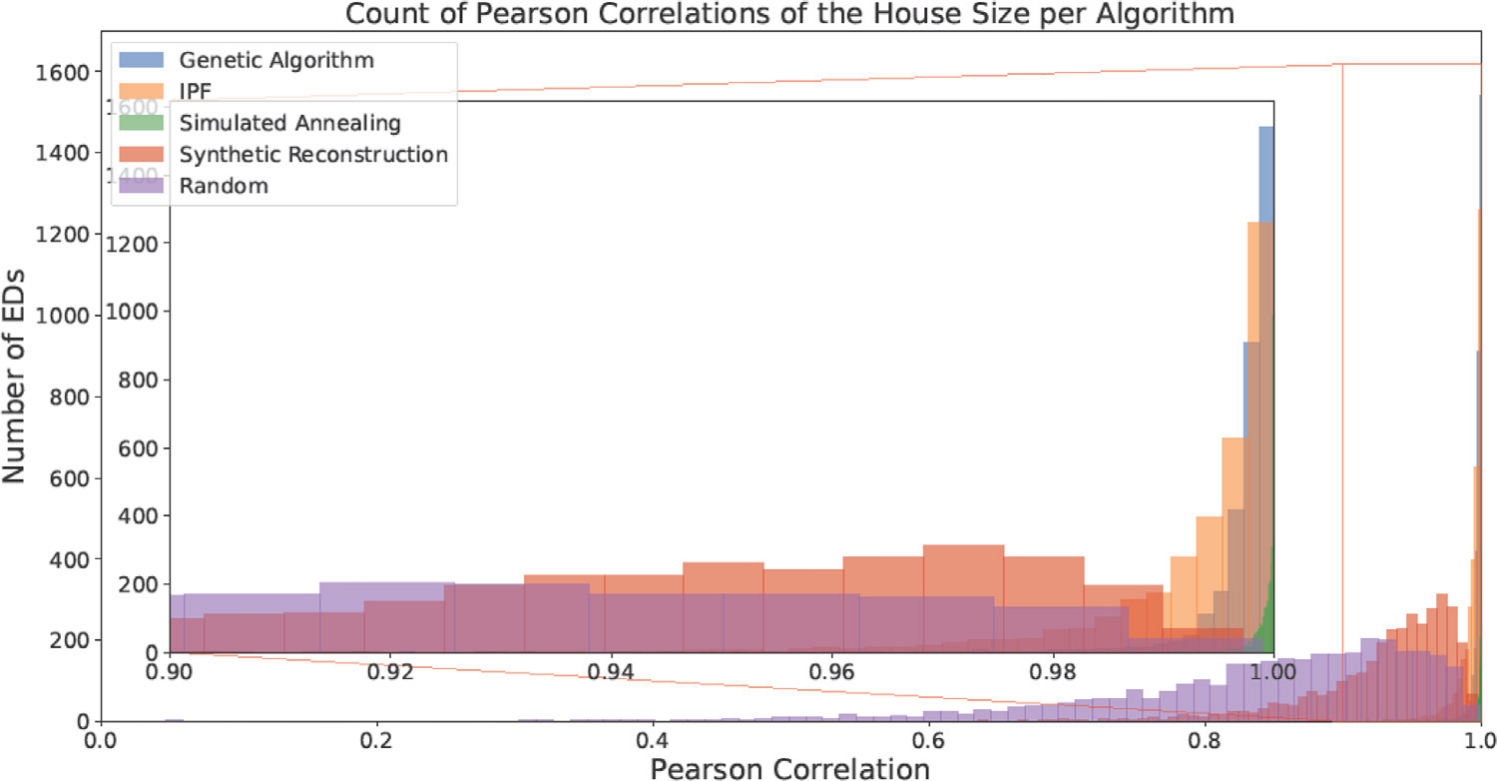
Counts of the Pearson Correlation per algorithm for House Size(with a focus on the area with > 0.9 correlation).

**Fig. 8:**
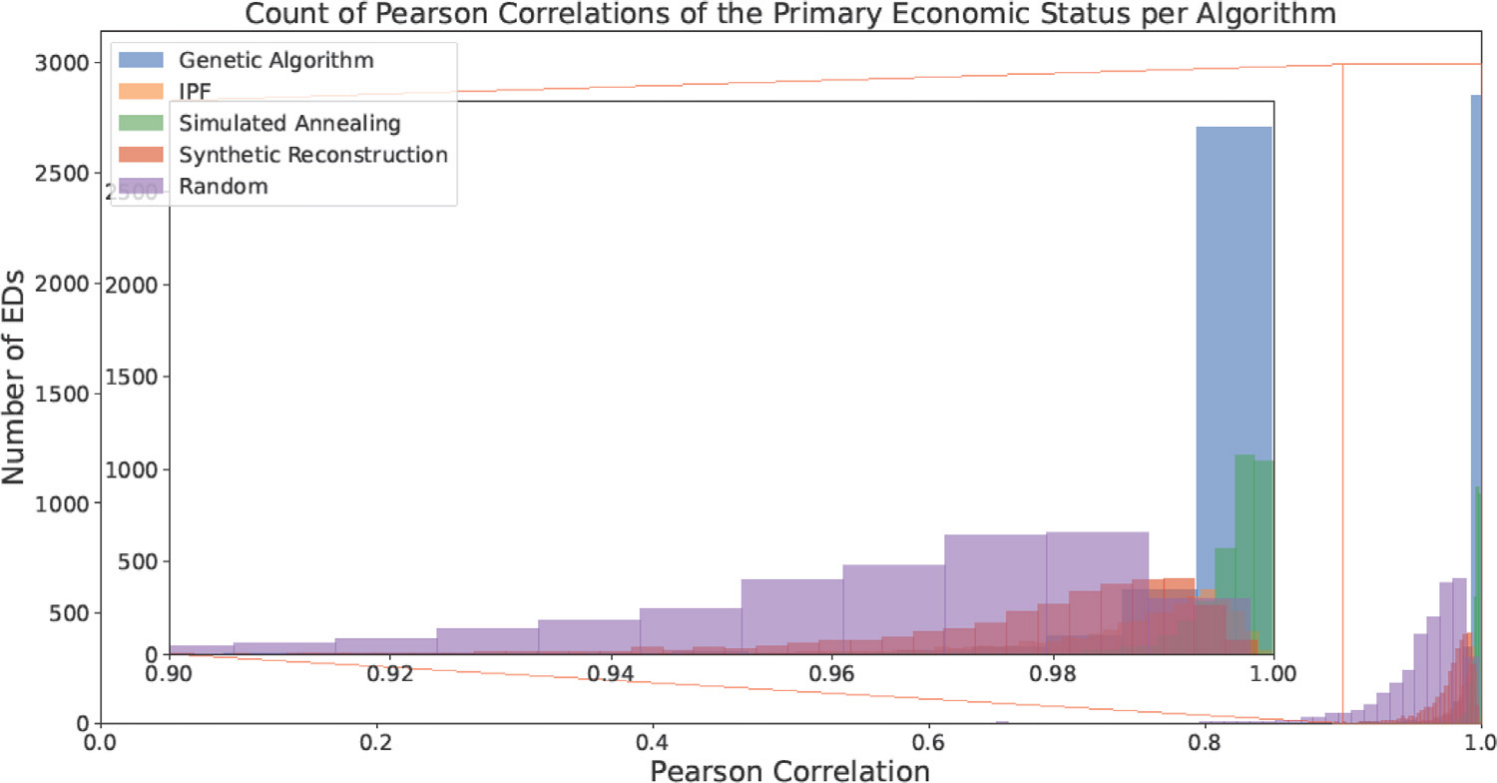
Counts of the Pearson Correlation per algorithm for Primary Economic Status (with a focus on the area with > 0.9 correlation).

**Fig. 9:**
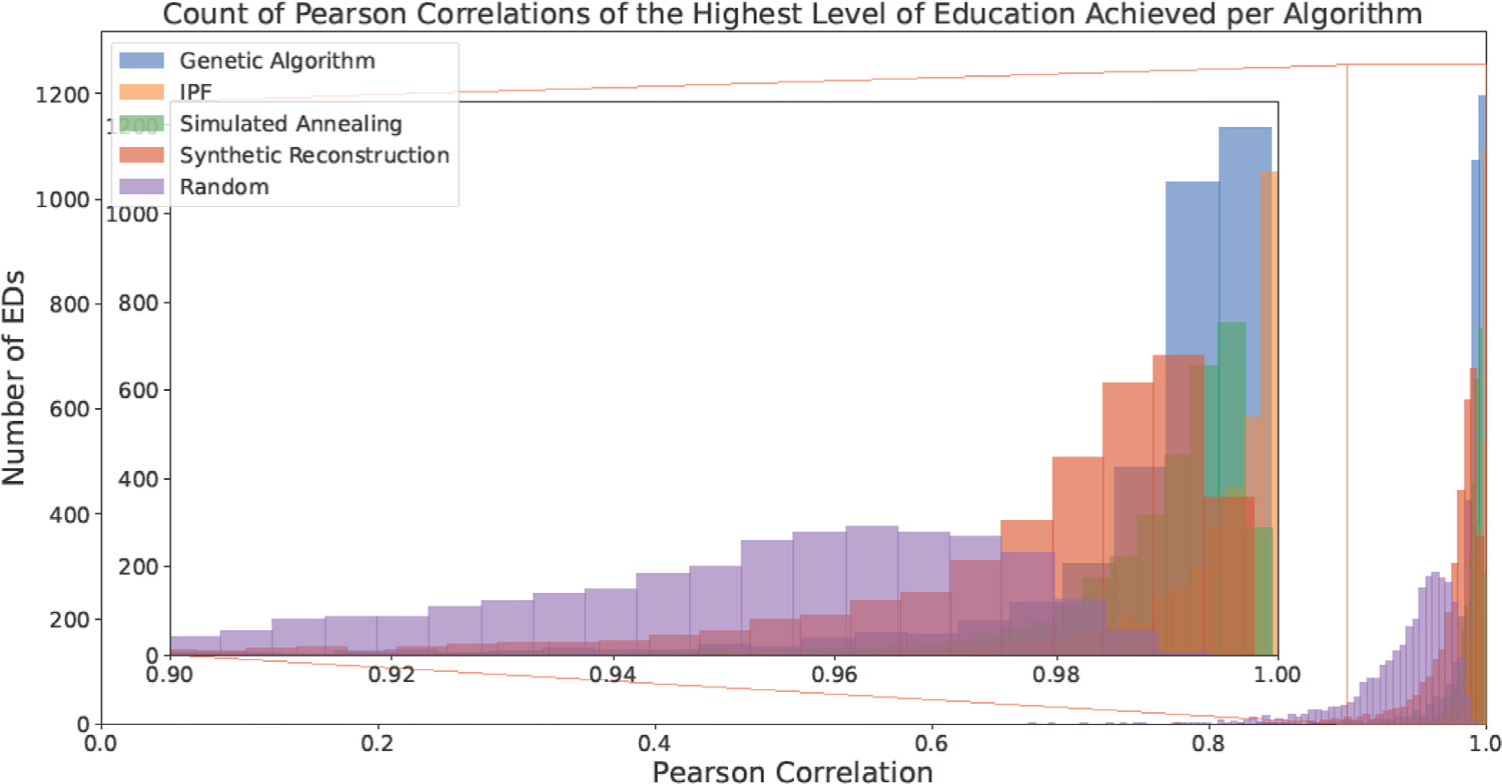
Counts of the Pearson Correlation per algorithm for Highest Level of Education Achieved (with a focus on the area with > 0.9 correlation).

Median results are a useful indicator of the average goodness-of-fit of the populations but to truly understand the distribution of fit, more detailed analysis must be performed. The counts of EDs' Pearson correlations for each characteristic are given in Fig. 5, Fig. 6, Fig. 7, Fig. 8, Fig. 9. All of the approaches (even including Random Selection) achieve acceptable results for the characteristics other than Age and Sex. In the case of the Age and Sex characteristic, IPF generates the most populations with the highest levels of fitness. These particularly accurate EDs (like the 0 NFT EDs outlined in the Evaluation at the Electoral Division Level Section) could be used as case studies for simulations where the maximum transferability to the real population was desired. The Genetic Algorithm approach presents a peak at an R-value of approximately 0.95. This implies that although GAs may not achieve a maximum correlation as high as other approaches, they can model a large proportion of EDs accurately (>0.9) without having many EDs below that threshold.

## Limitations

One of the key limitations of synthetic population generation influenced by microdata is that there may be real individuals whose combination of characteristics are not represented in the relevant survey, also known as the empty cell problem. This is inevitable when a survey of approximately 30 thousand individuals is being used to represent a national population of over 5 million. A limitation pertinent to the Irish case is that sometimes there is not a one-to-one correspondence between characteristics from the aggregate data and characteristics from the microdata. As outlined in the Input Data Section, every effort has been made to achieve a strong match between characteristics from both sides. However, there could be small discrepancies for characteristics such as Highest Level of Education Achieved, where the NFQ levels given in the SAPS data do not have an exact correspondence to the ISCED levels given in the LFS microdata.

One limitation in terms of the methodology itself is the integerisation of the weights generated by the IPF method. As IPF generates fractional weights, some type of rounding needs to be implemented to generate whole individuals for a population. The “truncate, replicate, sample” method, introduced by Lovelace et al. [10], which has been shown to be more accurate than simple rounding was used here. However, this method can still lead to mismatches between the marginal distributions of the real and simulated populations. Therefore, a user looking for the best possible accuracy could consider an integerisation process such as Quasirandom Integer Sampling. With regard to validation of the population; if a user had a particular use case for the population in mind, external validation by estimating some extra characteristics of the synthetic population could reveal the goodness-of-fit of the population for that use case. For example, a user wishing to perform simulations of income could use the economic status and education level attributes to predict each person in the synthetic population's income. Then, if statistics for the average income in Electoral Division was available, the average incomes of the synthetic population could be compared to these real averages. Another limitation is that while most of the approaches outlined above achieved a good fit to the aggregate statistics, there were no perfect matches overall. Therefore, it is advised that users of this data bear in mind these potential limitations at all times, and ensure results are corroborated by analytical tests as well as common sense.

## Ethics Statement

The authors confirm that this work doesn't not involve human subjects, animal experiments, or any data collected from social media platforms.

## Data Availability

Zenodo/GitHubA Synthetic Population Dataset for Ireland (Original data).

## Appendix Supplementary materials

Image, application 1

## Footnotes

1 Available from https://data.cso.ie/ - Census/SAPMAP 2022.
